# CTLA4 has a profound impact on the landscape of tumor-infiltrating lymphocytes with a high prognosis value in clear cell renal cell carcinoma (ccRCC)

**DOI:** 10.1186/s12935-020-01603-2

**Published:** 2020-10-27

**Authors:** Shiyi Liu, Feiyan Wang, Wei Tan, Li Zhang, Fangfang Dai, Yanqing Wang, Yaqi Fan, Mengqin Yuan, Dongyong Yang, Yajing Zheng, Zhimin Deng, Yeqiang Liu, Yanxiang Cheng

**Affiliations:** 1 Department of Obstetrics and Gynecology, Renmin Hospital of Wuhan University, Wuhan, 430060 Hubei Province People’s Republic of China; 2 Shanghai Skin Disease Clinical College of Anhui Medical University, Shanghai Skin Disease Hospital, Shanghai, 200443 People’s Republic of China; 3 Department of Dermatopathology, Shanghai Skin Disease Hospital, Tongji University School of Medicine, Shanghai, 200071 Shanghai People’s Republic of China; 4 Department of Dermatopathology, Shanghai Skin Disease Clinical College of Anhui Medical University, Shanghai Skin Disease Hospital, Shanghai, 200071 Shanghai People’s Republic of China

**Keywords:** CTLA4, Clear cell renal cell carcinoma, Tumor microenvironment, Tumor-infiltrating lymphocytes, Prognosis, CD8+ T cells, Immune checkpoints

## Abstract

**Background:**

Cytotoxic T-lymphocyte associated protein 4 (CTLA4) inhibitors have been shown to significantly prolong the overall survival (OS) in a wide range of cancers. However, its application in clear cell renal cell carcinoma (ccRCC) is limited due to the therapy response, and the prognostic value of CTLA4 in ccRCC has not been investigated in detail.

**Methods:**

By using immunohistochemistry, Kaplan–Meier (K–M) analysis, uni- and multi-variate Cox analysis, we comprehensively and systematically studied the prognostic value of CTLA4 in ccRCC. Then, we applied Gene Ontology (GO), the Kyoto Encyclopedia of Genes and Genomes (KEGG) and CIBERSORT, ESTIMATE algorithm, ssGSEA and somatic mutation analyses to reveal the impact of CTLA4 on the landscape of tumor-infiltrating lymphocytes (TILs) infiltration and genetic mutation. Besides, given current concerns caused by combined immunotherapy, we also investigated the relationship between CTLA4 and other immune checkpoints.

**Results:**

In vitro experiment and data mining showed that, CTLA4 was up-regulated in ccRCC tissues and closely related to the disease progression as well as a poor prognosis. Deeper researches demonstrated that CTLA4 regulates T cell activation and was significantly linked to TIL-abundant tumor microenvironment (TME), but was accompanied by an immunosuppressed phenotype. Mutation analysis showed that CTLA4 was associated with more frequent BRCA-associated protein 1 (BAP1) mutation. Moreover, we found that CTLA4 was markedly correlated with multiple immune checkpoints, which suggested that ccRCC patients with high expressed CTLA4 may benefit more from immune checkpoint blockades (ICBs) combined therapy.

**Conclusion:**

CTLA4 has a profound impact on the landscape of TILs and genetic mutation, and can be used as the biomarker with high prognosis value in ccRCC.

## Background

Clear cell renal cell carcinoma (ccRCC) is the most common and fatal histological subtype of renal cell carcinoma in adults, accounting for about 65–70% in RCC, characterized by abundant tumor-infiltrating lymphocytes (TILs) infiltration within the tumor microenvironment (TME) [1, 2]. Since the symptoms were not obvious, 20% of patients were initially diagnosed with metastases and nearly 30% relapsed with metastasis after surgical excision [3]. Therefore, the development of novel therapeutic strategies for ccRCC is necessary.

Along with advances in cancer immunology, the role of TME in ccRCC has attracted increased attention in recent years, consisting of cancer cells, fibroblasts, myofibroblasts, endothelial cells, TILs, and extracellular matrix [4]. The composition of TILs within TME determines whether its phenotype is anti-tumor immunity or immune evasion. Anti-tumor immunity is characterized by CD8+ T cells, M1 macrophages, while immune evasion is characterized by mast cells, T cells regulatory (Tregs), and M2-macrophages. Macrophages in tumor tissues are biased towards M2 subtype [5]. Furthermore, immune checkpoint within the TILs is a crucial factor in maintaining immune evasion of TME [6]. E.g. Tregs can inhibit the activation of CD8+ T cells through Cytotoxic T-lymphocyte associated protein 4 (CTLA4), triggering tumor immunosuppression [7]. Numerous clinical studies have reported that the immune checkpoint would be an ideal target for driving T cell mediated anti-tumor immunity [8, 9]. With tremendous progress, immune checkpoint blockades (ICBs) such as programmed death-ligand 1 (PD-L1) inhibitor Durvalumab and CTLA4 inhibitor Tremelimumab et al. have been shown to significantly prolong overall survival (OS) in a wide range of cancers [10–12].

CTLA4, immune checkpoint protein, has received extensive attention in immunotherapy. CTLA4 was highly expressed in CD8+ T cells and inhibited the T cells activation through competitively blocking the binding of CD28 with B7, leading to immune evasion [13]. Since the CTLA4 inhibitor ipilimumab (the first ICB approved by the FDA) has significantly prolonged the OS of patients with metastatic melanoma, CTLA4 inhibitors have proven to be effective agents for many cancers [14]. However, the clinical application of CTLA4 inhibitors in ccRCC is strictly limited, for that the therapeutic response and prognostic value of CTLA4 in ccRCC have not been investigated in detail. In the current work, we attempted to comprehensively analyze the prognostic value of CTLA4 in ccRCC and its impact on TILs and genetic landscape through bioinformatics and in vitro experiment, which may be beneficial to the therapeutic response of CTLA4 inhibitors in ccRCC patients.

## Materials and methods

### Data

The mRNA-seq data from 533 ccRCC and 72 normal tissues and corresponding clinical information, as well as 336 ccRCC somatic mutation data, were retrieved from The Cancer Genome Atlas (TCGA, https://portal.gdc.cancer.gov/), patients with OS more than 30 days were retained. Ultimately, 494 ccRCC and 68 normal tissues were included for our analyses. RNA-array data GSE40435 (GPL10558) and GSE46699 (GPL570) were obtained from the Gene Expression Omnibus (GEO, https://www.ncbi.nlm.nih.gov/geo/), including 101 ccRCC and paired normal kidney tissues, 67 ccRCC and 63 normal tissues, respectively.

### Immunohistochemistry

Formalin Fixed Paraffin Embedded (FFPE) ccRCC (n = 5) and corresponding normal specimens (n = 5) were collected from the department of pathology, Renmin Hospital of Wuhan University (China). Our study was approved by the Ethics Committee of Renmin Hospital of Wuhan University.

Immunohistochemistry with the rabbit monoclonal to CTLA4 (abcam, Shanghai, China) and the secondary antibody (Aspen, Wuhan, China) was performed following the manufacturer’s instructions [15]. Images were obtained using a BX63 microscope (Olympus, Japan), and the density of CTLA4 expression was evaluated by the percentage of CTLA4 positive cells in the total cells.

### Analysis of the functions of CTLA4

First, the CTLA4 related genes were screened based on Spearman correlation method with the absolute value of the correlation coefficient > 0.6 and *p* value < 0.05 as the threshold. Then, Gene Ontology (GO) and the Kyoto Encyclopedia of Genes and Genomes (KEGG) analyses were carried out through R package clusterProfiler [16] to reveal the potential function of CTLA4 in ccRCC.

### Genetic mutation analysis

The somatic mutation data of ccRCC patients from TCGA were analyzed by R package Maftool [17] to identify the impact of genetic mutation by CTLA4.

### Immune landscape of TME

CIBERSORT was a gene-based deconvolution algorithm developed by Newman et al. [18] and was applied to predict the abundance of immune cells using complex gene expression data in this investigation. Furthermore, the immune score was calculated by ESTIMATE algorithm, and the immunosuppression score was obtained from ssGSEA [19, 20].

### Statistics

Data analysis was conducted by using R 3.6.2 together with Graphpad prism 6. Difference of continuous variables between the two groups was calculated by the Wilcoxon test or student’s t test according to Shapiro-test and Bartlett-test. Chi-square (χ^2^) test was applied to assess the difference between categorical variables. Kaplan–Meier (K–M) analysis (Log-rank test based), uni- and multi-variate Cox analyses were used to evaluate the survival prognosis. Spearman method was utilized in correlation analysis. Data are presented as mean ± SD and *P* value < 0.05 was considered statistically significant (*p* < 0.05*, *p* < 0.01**, *p* < 0.001***).

## Result

### High prognosis value of CTLA4 in ccRCC

**Fig. 1:**
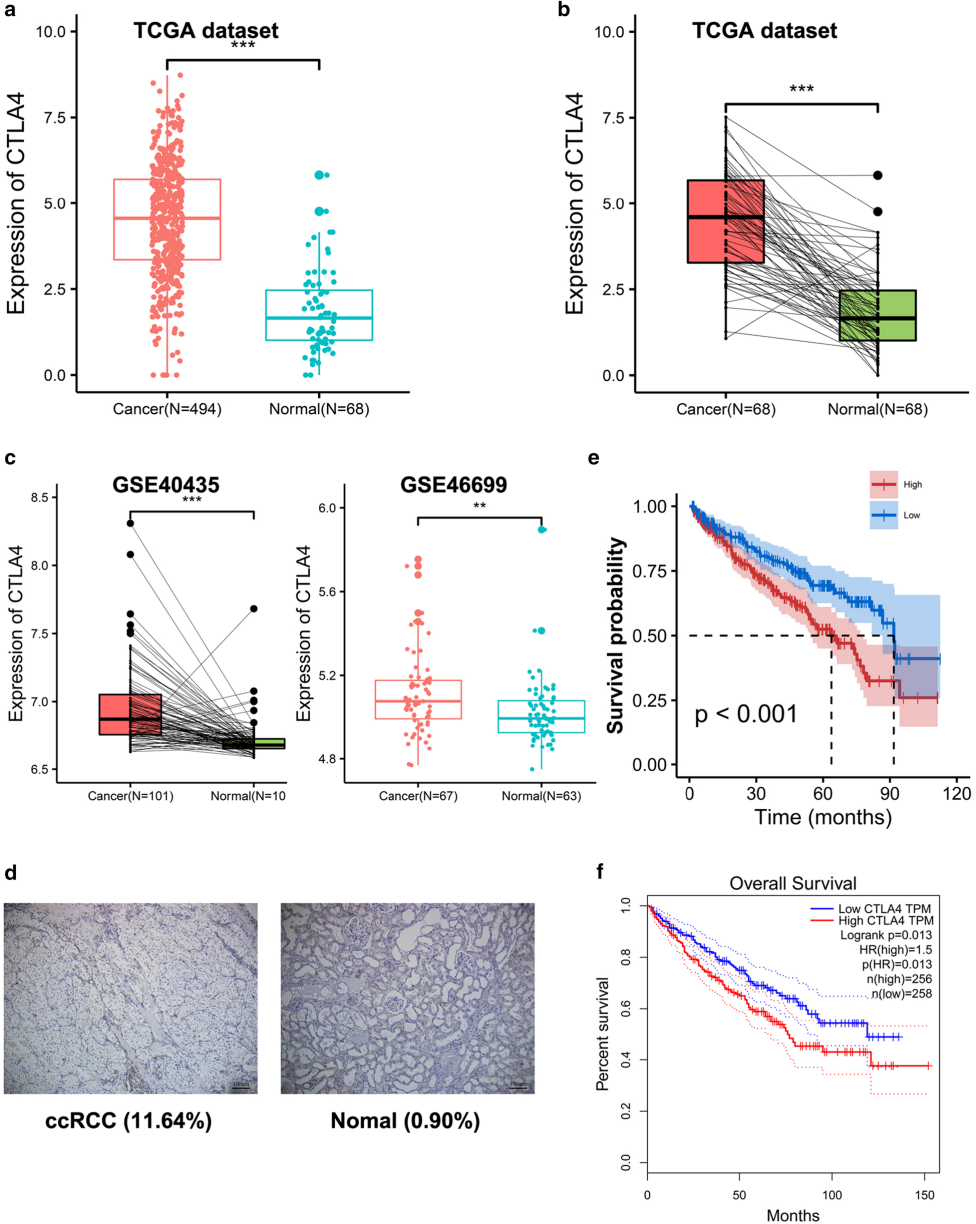
CTLA4 has a high prognosis value in ccRCC. The expression of CTLA4 between ccRCC and normal renal tissues in **a** TCGA dataset and **c** GSE46699. The expression of CTLA4 between paired ccRCC and normal tissues in **b** TCGA dataset and **c** GSE40435. **d** Verifying the expression of CTLA4 in ccRCC tissues through immunohistochemistry. Scale bars, 100 mm. Survival analysis in ccRCC patients from **e** TCGA dataset and **f** GEPIA database. ccRCC

In TCGA dataset, CTLA4 was highly expressed in tumor tissues (N = 494) compared to normal tissues (N = 68, *p* < 0.001), and the paired test also confirmed that CTLA4 was highly expressed in ccRCC (N = 68, *p* < 0.001) (Fig. 1a, b). Moreover, CTLA4 overexpression in ccRCC had also been reproduced in the GEO datasets GSE40435 and GSE46699 (Fig. 1c).

The immunohistochemistry was utilized to further validate the above results, and the result showed that the density of CTLA4 expression was higher in ccRCC tissues compared with normal tissues (*p* < 0.001, Fig. 1d). It seems that CTLA4 was steadily up-regulated in ccRCC both in data mining and in vitro experiment.

In TCGA dataset, patients were initially classified into high and low groups based on CTLA4 level. The K-M curves showed that CTLA4 shortened the OS in ccRCC, with the median survival time being 63.73 and 91.73 months in high and low group, respectively (*p* < 0.001) (Fig. 1e). In addition, 512 cases from GEPIA also confirmed that CTLA4 was a risk gene with Hazard ratio (HR) > 1 (HR = 1.5, *p* = 0.013, Fig. 1f).

We next sought to investigate the role of CTLA4 in cancer progression, and the result suggested that the overexpressed CTLA4 was related to high grade (χ^2^ = 12.465, *p* < 0.001), advanced stage (χ^2^ = 22.510, *p* < 0.001), patient with tumor state (χ^2^ = 7.874, *p* = 0.005) and death (χ^2^ = 9.965, *p* = 0.002, Table 1). We also found that CTLA4 had a positive correlation with high grade in GSE40435 cohorts (χ^2^ = 3.971, *p* = 0.046), indicating that CTLA4 may function as an oncogene in the progress of ccRCC. Subsequently, uni- and multi-variate Cox analyses found that some variables, including CTLA4, age, tumor pathological grade and stage, were independent risk factors in ccRCC (*p* < 0.01) (Table 2). These results proved that CTLA4 contributed to the progression of ccRCC with a high prognosis value.

**Table 1:** Correlation between CTLA4 and clinicopathological features in ccRCC

Variables	CTLA4^low^ group	CTLA4 ^high^ group	χ2	*P* value
Age				
< 60	119	113	0.244	0.622
≥ 60	127	134		
Gender				
Female	97	72	3.971	0.046
Male	150	175		
Grade				
1–2	126	89	12.465	< 0.001
3–4	114	157		
Stage				
I–II	175	122	22.510	< 0.001
III–IV	71	123		
Tumor status				
Negative	177	149	7.874	0.005
Positive	55	84		
Vital status				
Alive	183	150	9.965	0.002
Death	62	96

**Table 2:** Univariate and multivariate Cox analyses

Variables	Univariate cox regression		Multivariate cox regression	
	*p* value	HR (95% CI)	*p* value	HR (95% CI)
CTLA4	6.26e−08	1.30 (1.40–1.70)	0.00184	1.22 (1.13–1.41)
Age	0.000661	1.80 (1.30–2.60)	0.00948	1.58 (1.12–2.24)
Gender	0.788	1.00 (0.76–1.40)	*NA*	*NA*
Grade	4.36e−08	2.80 (1.90–4.0)	0.00279	1.80 (1.22–2.56)
Stage	5.56e−17	4.20 (3.00–5.90)	1.55e−09	3.02 (2.11–4.32)
LDH_level	0.464	1.50 (0.51–4.40)	*NA*	*NA*

### CTLA4 indicated a higher density of TILs in ccRCC tumor microenvironment, but an immunosuppressed phenotype.

**Fig. 2:**
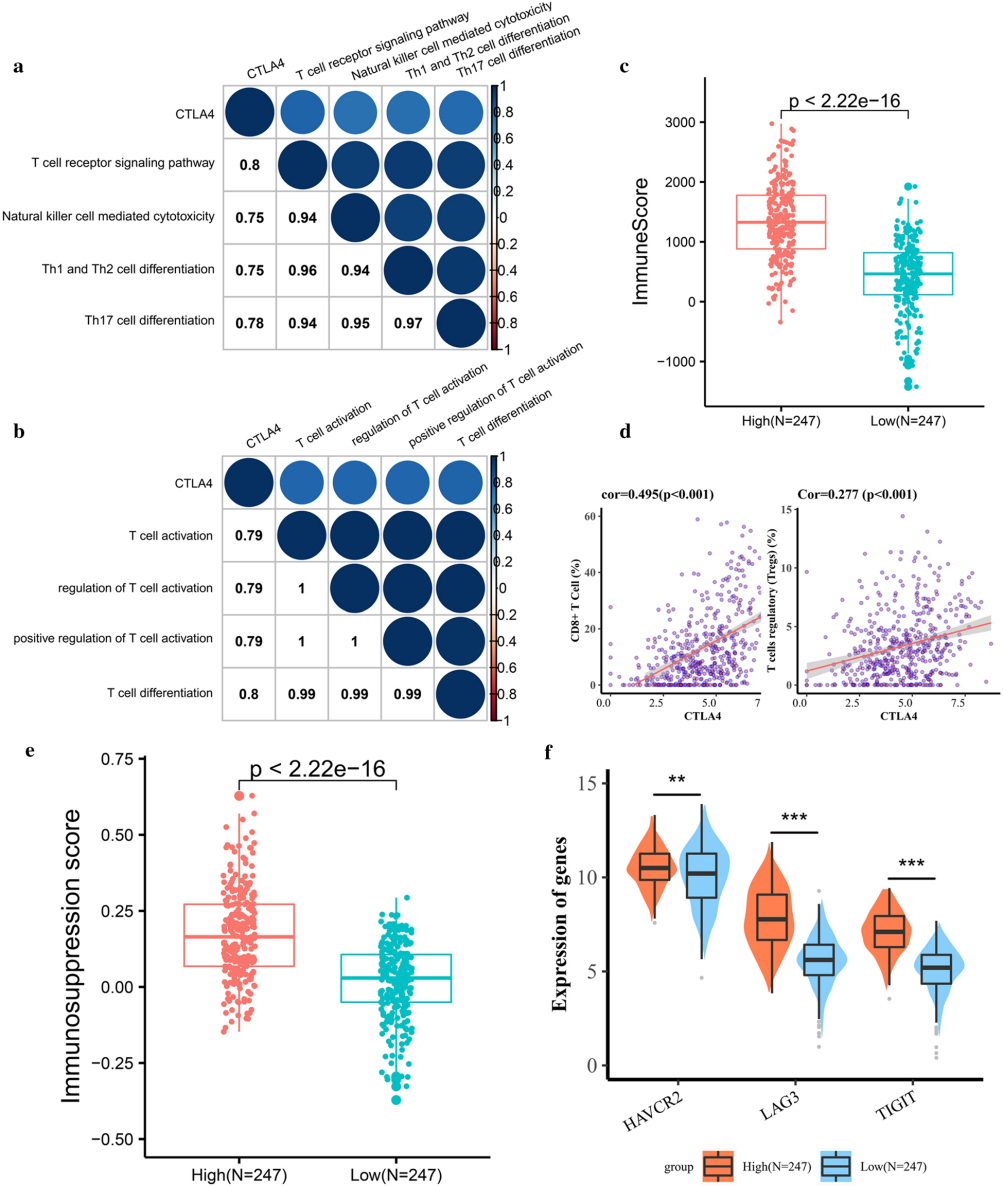
CTLA4 indicated a higher density of TILs in ccRCC tumor microenvironment, but an immunosuppressed phenotype. **a** CTLA4 is positively correlated with T cell receptor signaling pathway, Natural killer cell mediated cytotoxicity, Th1 and Th2 cell differentiation and Th17 cell differentiation. **b** CTLA4 is positively correlated with T cell activation and T cell differentiation. **c** Highly expressed CTLA4 has a higher Immune score, indicating that there are more TILs within the TME. **d** CTLA4 is positively correlated with infiltrating CD8+ T cells and Tregs. **e**, **f** Highly expressed CTLA4 is accompanied by a higher Immunosuppression score and higher expression of T cell exhaustion markers, indicating an immunosuppressed phenotype. TILs, tumor-infiltrating lymphocytes; TME, tumor microenvironment; Tregs, T regulatory cells

To outline the corresponding function of the CTLA4 in ccRCC TME, we performed KEGG and GO analysis based on 200 CTLA4 related protein coding genes (Additional file 1: Table S1). 35 KEGG items were identified, including T cell mediated immune related pathway, natural killer cell mediated cytotoxicity, T helper cells differentiation, PD-L1 expression and programmed death‐1 receptor (PD-1) checkpoint pathway in cancer (Table 3), and CTLA4 was positively correlated with T cell receptor signaling pathway (cor = 0.80), Natural killer cell mediated cytotoxicity (cor = 0.75), Th1 and Th2 cell differentiation (cor = 0.75) and Th17 cell differentiation (cor = 0.78) (Fig. 2a). The biological processes of CTLA4 yielded from the GO analysis were associated with the activation and differentiation of T cells (Table 3) and positively correlated with T cell activation (cor = 0.79), regulation of lymphocyte activation (cor = 0.79) and T cell differentiation(cor = 0.80) (Fig. 2b).

**Table 3:** Top 10 items in functional enrichment analysis

KEGG	GO (BP)
T cell receptor signaling pathway	T cell activation
Primary immunodeficiency	Regulation of T cell activation
Natural killer cell mediated cytotoxicity	Leukocyte cell–cell adhesion
Th1 and Th2 cell differentiation	Regulation of lymphocyte activation
Th17 cell differentiation	Regulation of leukocyte cell–cell adhesion
Cell adhesion molecules (CAMs)	Positive regulation of T cell activation
Chemokine signaling pathway	Regulation of cell–cell adhesion
PD-L1 expression and PD-1 checkpoint pathway in cancer	T cell differentiation
Hematopoietic cell lineage	Positive regulation of leukocyte cell–cell adhesion
Human immunodeficiency virus 1 infection	Positive regulation of cell–cell adhesion

Previous studies have demonstrated that TILs within the TME can be regarded as a prognostic indicator in ccRCC [21]. Besides, the results of GO and KEGG promoted us to continue to investigate the role of CTLA4 in TILs infiltration, which affected the ICBs’ response. Our results showed that CTLA4 was associated with a higher immune score, which was calculated by the ESTIMATE algorithm and represented the level of TILs, indicating that CTLA4 promoted the recruitment of immune cells into the TME (Fig. 2c). Furthermore, there was a great difference in the composition of TILs between high and low CTLA4 groups. CTLA4 increased the infiltration of T Cells CD8+ , Tregs, Macrophage M1, whereas Plasma cells, NK cells activated, Monocytes, Macrophage M2, Dendritic cells activated were less infiltrated in CTLA4 high group (Additional file 1: Table S2). The correlation analysis result was presented in Fig. 2d, showing that CTLA4 was positively correlated with CD8+ T cells (cor = 0.50, *p* < 0.001), Tregs (cor = 0.28, *p* < 0.001) (Fig. 2d). However, the immunosuppression score as well as the expression of CD8 + T cell exhaustion markers Hepatitis A virus cellular receptor 2 (HAVCR2), lymphocyte activation gene-3 (LAG3), and T cell immunoglobulin and ITIM domain (TIGIT) was higher in CTLA4 high group (Fig. 2e, f). All in all, CTLA4 changed the landscape of TILs in ccRCC TME, and indicated a higher density of TILs, especially the CD8+ T cells and Tregs, but faced an immunosuppressed phenotype.

### Genetic altered by CTLA4 in ccRCC

**Fig. 3:**
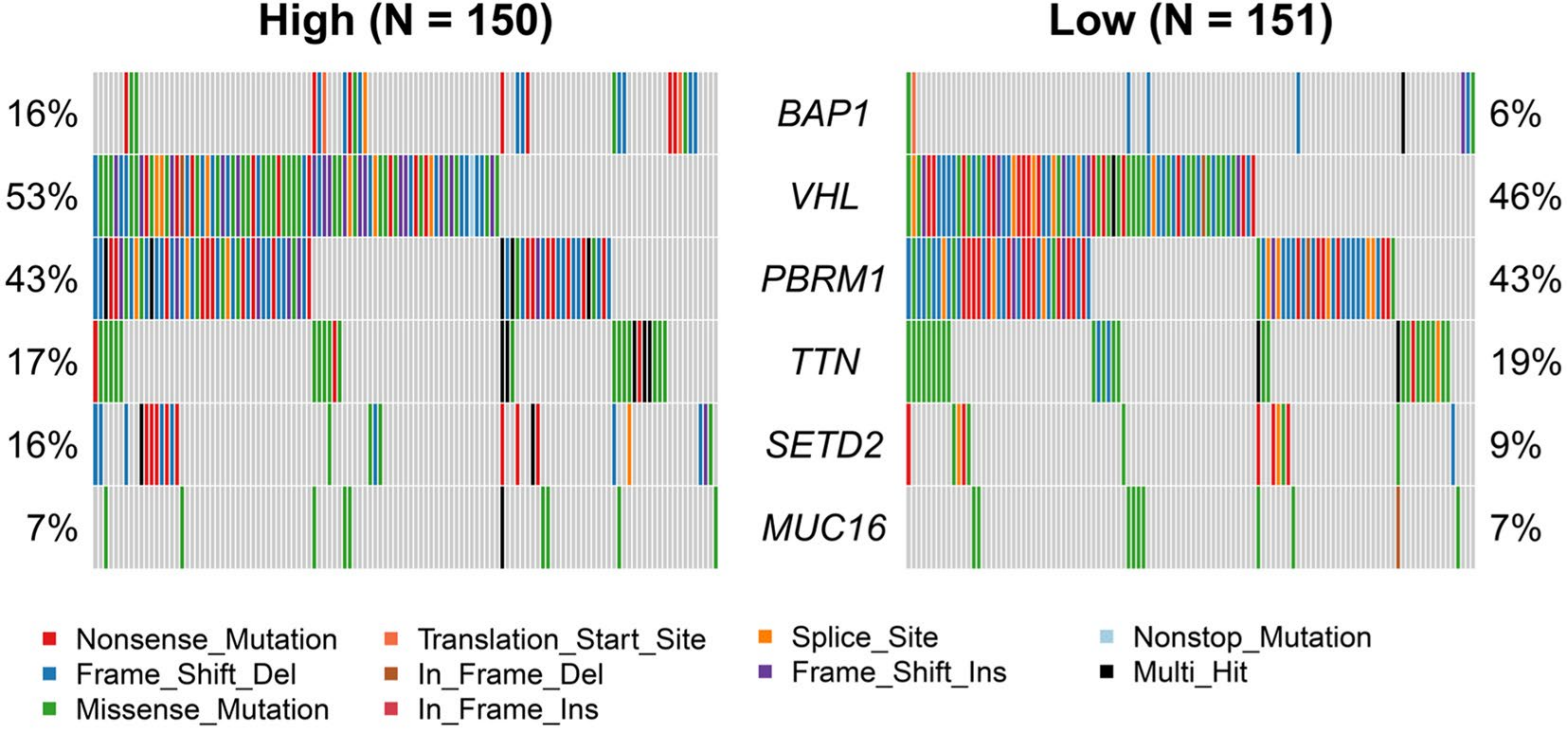
Genetic changes by CTLA4 in ccRCC. Genetic mutation analysis of CTLA4 between high and low CTLA4 groups, the result shows that the BAP1 mutation is more frequent in the CTLA4 high group

Genetic changes include non-synonymous mutations, which are mainly composed of missense mutation, synonymous mutation, insertion or deletion, and copy number gain or loss [22–25]. Tumor mutation burden (TMB) can be used as a biomarker to predict the efficacy of ICBs [26]. Some studies have shown that the RCC was sensitive to ICBs, although the TMB in RCC was moderate [27, 28]. To identify the somatic mutations that were altered by CTLA4 in ccRCC, we performed the mutation analysis and the result showed that overexpressed CTLA4 was correlated with BRCA-associated protein 1 (BAP1) mutation (*p* < 0.05, Fig. 3). The TMB in the high CTLA4 expression group tended to be higher than the low expression group, although it was not statistically significant. Moreover, Nonsense Mutation and In Frame Ins in the high CTLA4 expression group were higher than those in the low group (Table 4). BAP1 is a deubiquitinating enzyme and considered to be a tumor suppressor, and the loss of BAP1 contributes to the metastasis and poor prognosis in various cancers [29].

**Table 4:** Classification of variants in different groups

Type of variants	High group (n = 56)		Low group (n = 63)	
	Summary	Mean	Summary	Mean
Frame Shift Del	782	5.21	791	5.24
Frame Shift Ins	938	6.25	211	1.4
In Frame Del	97	0.65	114	0.75
In Frame Ins*	331	2.21	13	0.09
Missense mutation	5922	39.48	5783	38.3
Nonsense mutation**	772	5.15	385	2.55
Nonstop mutation	8	0.05	9	0.06
Splice site	212	1.41	223	1.48
Translation start site	14	0.09	9	0.06
Total	9076	60.51	7542	49.95

wilcox.test * *p* < 0.05, ** *p* < 0.01

### CTLA4 was highly related to other immune checkpoint molecules

**Fig. 4:**
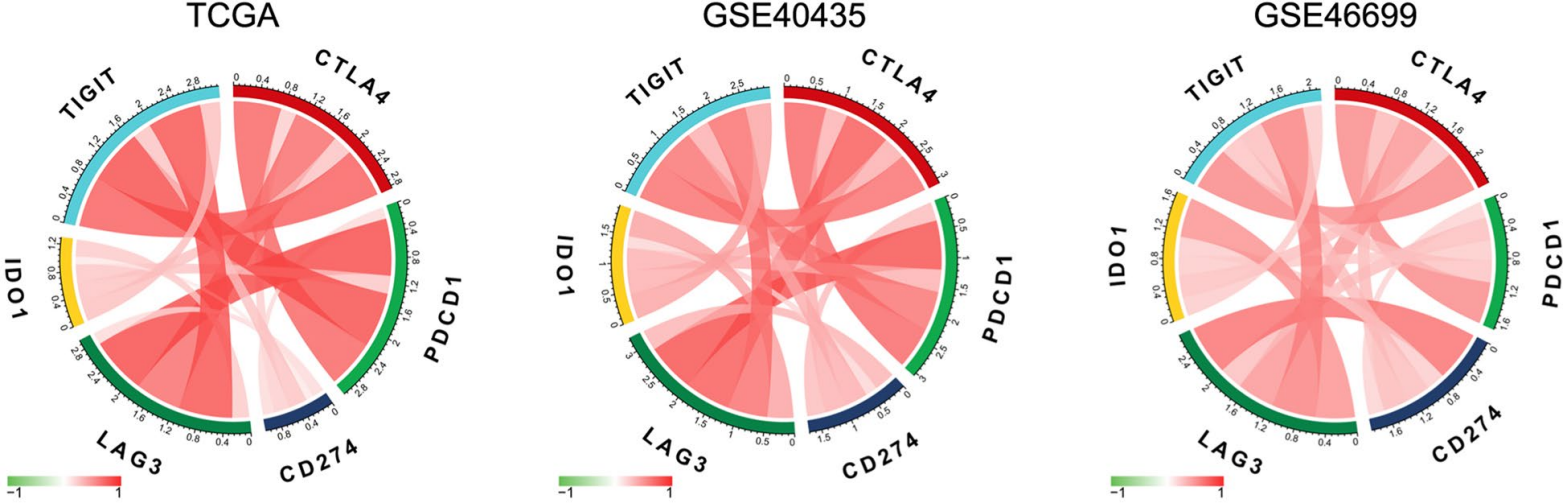
CTLA4 was highly related to other immune checkpoint molecules. CTLA4 is highly and positively related to other immune checkpoint molecules PDCD1(PD-1), CD274(PD-L1), LAG3, IDO1, TIGIT

Recently, the combined inhibition of PD-L1 and CTLA4 has attracted much attention [30]. Planchard et al. [31] reported that combination immunotherapy of PD-L1 and CTLA4 considerably prolonged the OS in advanced refractory colorectal cancer. Combination immunotherapy tends to replace monotherapy, for that the combinational usage of ICBs can produce higher synergistic anti-tumor efficiency and reduce side effects [32]. Therefore, we continued to explore the correlation between CTLA4 and other immune checkpoint molecules, including PDCD1 (PD-1), CD274 (PD-L1), LAG3, indoleamine-2,3-dioxygenase-1 (IDO1), and TIGIT [33, 34]. The results showed that CTLA4 was highly and positively related to PD-1, PD-L1, LAG3, IDO1, and TIGIT (Fig. 4).

## Discussion

CTLA4, as a transmembrane protein expressed in activated CD4+ T and CD8+ T cells, has received a lot of attention for its interaction with cancer. CTLA4 negatively regulates T cell activation by blocking the function of costimulatory signal and differentiation cluster CD28:B7 binding [35]. CTLA4 inhibitors reverse inhibitory immune signal and restore the anti-cancer response by blocking the interaction between CTLA4 and the ligand expressed by antigen presenting cells [32]. With the approval of CTLA4 inhibitor Ipilimumab for clinical applications, it has been used for metastatic melanoma after the first-line treatment [14]. Furthermore, Romano et al. [36] proved that Ipilimumab can exert a therapeutic effect by targeting Tregs in tumors.

Several studies have shown that CD8+ T cells are in a state of abnormal activation when they turn to exhausted phenotypes due to the elimination of tumor cells, which can not only up-regulate the expression of immunosuppressive cytokines, but also directly lead to immunosuppression [37]. Exhausted CD8+ T cells continue to activate the expression of CTLA4 and other immune checkpoint receptors under the chronic stimulation of tumor antigens, which further promoting tumor invasion [38]. Here, we confirmed that CTLA4 was up-regulated in ccRCC tissues through data mining and in vitro experiment, and revealed that CTLA4 was correlated with poor prognosis. Corresponding to other studies, this study confirmed that CTLA4 played an important role in the regulation of T cells and represented more TILs infiltration to the TME, especially the CD8+ T cells and Tregs. However, the phenotype of the TME trended to immunosuppression, and the infiltrating CD8+ T cells biased to exhaustion in the CTLA4 high group, synergizing with Tregs, ultimately leading to tumor metastasis and progression.

We noticed that tumors with high TMB were sensitive to ICBs, contributing to a better outcome [26]. Therefore, we tried to outline the relationship between somatic mutation and CTLA4. We found that the Nonsense Mutation and In Frame Ins were markedly higher, and the BAP1 mutation was more frequent in the CTLA4 high group. As a tumor suppressor gene, the loss of BAP1 tended to cause poor prognosis and higher TMB, suggesting that patients with high CTLA4 expression might be more sensitive to ICBs. Furthermore, the somatic mutation of BAP1 is more abundant in highly metastatic tumors, such as uveal melanoma [39]. BAP1 is located at chromosome 3p21, adjacent to 3p25 where VHL is located [40]. Considering that 3p deletions in ccRCC are common, we think that 3p deletions might cause the inactivation of these tumor suppressor genes. Nevertheless, further explorations are needed to reveal the underlying mechanisms between CTLA4 and BAP1.

Finally, we revealed that CTLA4 was highly related to other immune checkpoints: PD-1, PD-L1, LAG3, IDO1, and TIGIT. Activation of the PD-1/PD-L1 signaling pathway contributes to TME with immune evasion, and its inhibitors are representative, which have been used in lots of solid tumors [41]. LAG3 can negatively regulate the activation and function of T cells, and its antagonists have been applied clinically [42]. At present, some studies have been devoted to the synergism between LAG3 and PD-1 in enhancing the efficacy of immunotherapy [43]. IDO1 is overexpressed in cancer cells, inhibiting the function of effector T cells and promoting the infiltration of Tregs. Studies have demonstrated that IDO1 is a promising target for improving patient outcomes in the field of immune- oncology [44]. The above results suggested that the CTLA4 inhibitor combined with other ICBs like PD-1 inhibitor nivolumab or LAG3 inhibitor may obtain a better therapeutic response in ccRCC, since that preclinical and clinical studies have provided evidence that combination inhibitor of CTLA4 and other ICBs can enhance the anti-tumor efficiency of CD8+ T cells [45, 46].

## Conclusion

We comprehensively and systematically studied the prognostic value of CTLA4 in ccRCC and its impact on the landscape of TME TILs infiltration and genetic mutation, finding that CTLA4, acted like an oncogene, can accelerate the progression of ccRCC with a high prognostic value, and that CTLA4 was associated with more TILs infiltrated TME but had an immunosuppressed phenotype. Besides, patients with high CTLA4 levels may benefit more from the combined ICBs therapy. However, the potential role of CTLA4 in the progression of ccRCC needs further verification in vitro and in vivo.

## Supplementary information

**Additional file 1: Table S1.** CTLA4-related protein coding genes by correlation analysis. **Table S2.** Comparison of CIBERSORT immune cell fractions.

## Data Availability

All analyzed data are included in this published article and its additional file. The original data used during the current study are available from the corresponding author on reasonable request.

## Abbreviations

CTLA4: Cytotoxic T-lymphocyte associated protein 4
ccRCC: Clear cell renal cell carcinoma
TME: Tumor microenvironment
TILs: Tumor-infiltrating lymphocytes
OS: Overall survival
BAP1: BRCA-associated protein 1
ICBs: Immune checkpoint blockades
Tregs: T cells regulatory
PD-1: Programmed death-1 receptor
PD-L1: Programmed death-ligand 1
TCGA: The Cancer Genome Atlas
GEO: Gene Expression Omnibus
FFPE: Formalin fixed paraffin embedded
GO: Gene Ontology
KEGG: Kyoto Encyclopedia of Genes and Genomes
K-M: Kaplan–Meier
HR: Hazard ratio
HAVCR2: Hepatitis A virus cellular receptor 2
LAG3: Lymphocyte activation gene-3
TIGIT: T cell immunoglobulin and ITIM domain
TMB: Tumor mutation burden
IDO1: Indoleamine-2,3-dioxygenase-1
