# Correction to: CTLA4 has a profound impact on the landscape of tumor-infiltrating lymphocytes with a high prognosis value in clear cell renal cell carcinoma (ccRCC)

**DOI:** 10.1186/s12935-021-02005-8

**Published:** 2021-06-07

**Authors:** Shiyi Liu, Feiyan Wang, Wei Tan, Li Zhang, Fangfang Dai, Yanqing Wang, Yaqi Fan, Mengqin Yuan, Dongyong Yang, Yajing Zheng, Zhimin Deng, Yeqiang Liu, Yanxiang Cheng

**Affiliations:** 1 Department of Obstetrics and Gynecology, Renmin Hospital of Wuhan University, Wuhan, 430060 Hubei People’s Republic of China; 2 Shanghai Skin Disease Clinical College of Anhui Medical University, Shanghai Skin Disease Hospital, Shanghai, 200443 People’s Republic of China; 3 Department of Dermatopathology, Shanghai Skin Disease Hospital, Tongji University School of Medicine, Shanghai, 200071 People’s Republic of China

## Correction to: Cancer Cell Int (2020) 20:519 https://doi.org/10.1186/s12935-020-01603-2

Following the publication of the original article [1], we were notified that the second and fourth affiliations are the same, so the first affiliation of Yeqiang Liu is changed to 2, and his second affiliation is changed to 3, as shown below:

^2^Shanghai Skin Disease Clinical College of Anhui Medical University, Shanghai Skin Disease Hospital, Shanghai, 200443, People’s Republic of China.

^3^Department of Dermatopathology, Shanghai Skin Disease Hospital, Tongji University School of Medicine, Shanghai, 200071, People’s Republic of China.
