# Supplementary material for: CTLA4 has a profound impact on the landscape of tumor-infiltrating lymphocytes with a high prognosis value in clear cell renal cell carcinoma (ccRCC)
Source: Cancer Cell Int. 2020 Oct 27;20:519. doi: 10.1186/s12935-020-01603-2 (PMC7590466; doi:10.1186/s12935-020-01603-2)
Supplement: Supplementary file 1 — Additional file 1: Table S1. CTLA4-related protein coding genes by correlation analysis. Table S2. Comparison of CIBERSORT immune cell fractions. [file 12935_2020_1603_MOESM1_ESM.docx]

**Table S1 CTLA4-related protein coding genes by correlation analysis**

| **genes** | **Correlation**  **coefficient** | **adj.*p*** | **genes** | **Correlation**  **coefficient** | **adj.*p*** |
| --- | --- | --- | --- | --- | --- |
| **SEPTIN1** | 0.794461 | 1.24E-108 | **IL2RG** | 0.751168 | 8.53E-91 |
| **SEPTIN6** | 0.609977 | 1.13E-51 | **IRF1** | 0.613555 | 2.02E-52 |
| **ABCA7** | 0.609994 | 1.13E-51 | **ITGAL** | 0.755097 | 2.94E-92 |
| **ACAP1** | 0.834136 | 3.14E-129 | **ITK** | 0.794609 | 1.06E-108 |
| **ADCY7** | 0.628104 | 1.43E-55 | **JAK3** | 0.643366 | 4.61E-59 |
| **ADRBK1** | 0.671264 | 5.51E-66 | **JAKMIP1** | 0.74174 | 2.15E-87 |
| **AKNA** | 0.805771 | 5.17E-114 | **KIF21B** | 0.731453 | 7.56E-84 |
| **APOBEC3D** | 0.741849 | 1.97E-87 | **KLHL6** | 0.641869 | 1.03E-58 |
| **APOBEC3G** | 0.66427 | 3.52E-64 | **KLRK1** | 0.776575 | 8.89E-101 |
| **APOBEC3H** | 0.670322 | 9.71E-66 | **LAG3** | 0.68071 | 1.67E-68 |
| **ARHGAP30** | 0.653809 | 1.44E-61 | **LCK** | 0.743932 | 3.59E-88 |
| **ARHGAP9** | 0.828718 | 4.15E-126 | **LCP2** | 0.642944 | 5.79E-59 |
| **ARHGEF1** | 0.67537 | 4.55E-67 | **LILRB1** | 0.604167 | 1.79E-50 |
| **BATF** | 0.709147 | 1.08E-76 | **LILRB2** | 0.620261 | 7.47E-54 |
| **BCL11B** | 0.60392 | 2.01E-50 | **LIMD2** | 0.719509 | 6.24E-80 |
| **BCL2L14** | 0.689189 | 7.58E-71 | **LOC100188949** | 0.826676 | 5.83E-125 |
| **BIN2** | 0.710366 | 4.55E-77 | **LOC100192379** | 0.634755 | 4.54E-57 |
| **BTLA** | 0.66262 | 9.24E-64 | **LOC100233209** | 0.714929 | 1.75E-78 |
| **BTN3A1** | 0.600018 | 1.24E-49 | **LOC389634** | 0.61882 | 1.53E-53 |
| **C15orf53** | 0.630691 | 3.77E-56 | **LPXN** | 0.658268 | 1.14E-62 |
| **C16orf54** | 0.620457 | 6.77E-54 | **LTA** | 0.718748 | 1.09E-79 |
| **C17orf66** | 0.738287 | 3.48E-86 | **LY9** | 0.620509 | 6.60E-54 |
| **C1orf200** | 0.621939 | 3.23E-54 | **LYST** | 0.61219 | 3.91E-52 |
| **C2orf85** | 0.758484 | 1.53E-93 | **MAP4K1** | 0.840341 | 6.04E-133 |
| **C5orf56** | 0.634612 | 4.89E-57 | **MCART6** | 0.690533 | 3.17E-71 |
| **C5orf58** | 0.651531 | 5.17E-61 | **MICAL1** | 0.679141 | 4.44E-68 |
| **CCDC88B** | 0.692569 | 8.37E-72 | **MIR155HG** | 0.694133 | 2.99E-72 |
| **CCL5** | 0.697306 | 3.62E-73 | **MYO1F** | 0.658658 | 9.14E-63 |
| **CCR5** | 0.668057 | 3.76E-65 | **NELL2** | 0.65692 | 2.47E-62 |
| **CD2** | 0.723295 | 3.77E-81 | **NKG7** | 0.617634 | 2.74E-53 |
| **CD244** | 0.64781 | 4.07E-60 | **NLRC3** | 0.735952 | 2.23E-85 |
| **CD247** | 0.700463 | 4.32E-74 | **NLRC5** | 0.667096 | 6.66E-65 |
| **CD27** | 0.700601 | 3.94E-74 | **NOD2** | 0.636905 | 1.46E-57 |
| **CD37** | 0.68042 | 2.00E-68 | **P2RY10** | 0.688821 | 9.61E-71 |
| **CD38** | 0.614232 | 1.45E-52 | **PARP15** | 0.671846 | 3.88E-66 |
| **CD3D** | 0.69714 | 4.05E-73 | **PARVG** | 0.800519 | 1.80E-111 |
| **CD3E** | 0.730061 | 2.22E-83 | **PATL2** | 0.721599 | 1.33E-80 |
| **CD48** | 0.624985 | 6.97E-55 | **PDCD1** | 0.653384 | 1.83E-61 |
| **CD5** | 0.706779 | 5.64E-76 | **PIK3CD** | 0.691988 | 1.22E-71 |
| **CD6** | 0.703454 | 5.62E-75 | **PLCB2** | 0.75194 | 4.42E-91 |
| **CD69** | 0.721891 | 1.07E-80 | **PPM1M** | 0.649708 | 1.42E-60 |
| **CD72** | 0.748358 | 9.14E-90 | **PSTPIP1** | 0.732547 | 3.23E-84 |
| **CD80** | 0.632989 | 1.14E-56 | **PTPN22** | 0.781377 | 8.20E-103 |
| **CD8A** | 0.664659 | 2.80E-64 | **PTPN6** | 0.630112 | 5.08E-56 |
| **CD8B** | 0.623045 | 1.86E-54 | **PTPN7** | 0.834395 | 2.22E-129 |
| **CD96** | 0.736267 | 1.74E-85 | **PTPRC** | 0.614674 | 1.17E-52 |
| **CEP110** | 0.626379 | 3.44E-55 | **PTPRCAP** | 0.649541 | 1.56E-60 |
| **CHFR** | 0.60303 | 3.05E-50 | **PVRIG** | 0.716262 | 6.69E-79 |
| **CIITA** | 0.679533 | 3.48E-68 | **PYHIN1** | 0.757184 | 4.78E-93 |
| **CLEC2D** | 0.625332 | 5.85E-55 | **RAC2** | 0.685559 | 7.80E-70 |
| **CLECL1** | 0.606315 | 6.49E-51 | **RASAL3** | 0.76784 | 3.35E-97 |
| **CORO1A** | 0.726413 | 3.61E-82 | **RASSF5** | 0.717123 | 3.58E-79 |
| **CRLF3** | 0.651226 | 6.12E-61 | **RGS1** | 0.67942 | 3.73E-68 |
| **CRTAM** | 0.678986 | 4.89E-68 | **RHOH** | 0.770304 | 3.40E-98 |
| **CST7** | 0.650968 | 7.07E-61 | **RIPK3** | 0.6161 | 5.83E-53 |
| **CTLA4** | 1 | 0 | **RLTPR** | 0.628646 | 1.08E-55 |
| **CXCL13** | 0.614633 | 1.19E-52 | **RRN3P2** | 0.680515 | 1.89E-68 |
| **CXCR3** | 0.651274 | 5.96E-61 | **SAMD3** | 0.688907 | 9.09E-71 |
| **CXCR6** | 0.688399 | 1.26E-70 | **SASH3** | 0.700586 | 3.98E-74 |
| **CXorf65** | 0.71137 | 2.24E-77 | **SCML4** | 0.715005 | 1.66E-78 |
| **CYTH4** | 0.616007 | 6.10E-53 | **SH2D1A** | 0.723443 | 3.37E-81 |
| **DEF6** | 0.776453 | 1.00E-100 | **SH2D2A** | 0.705267 | 1.61E-75 |
| **DGKA** | 0.686031 | 5.77E-70 | **SH3BP1** | 0.660223 | 3.71E-63 |
| **DTHD1** | 0.668889 | 2.29E-65 | **SIDT1** | 0.606341 | 6.41E-51 |
| **DUSP2** | 0.713226 | 5.96E-78 | **SIRPG** | 0.775957 | 1.61E-100 |
| **EVI2B** | 0.630237 | 4.76E-56 | **SIT1** | 0.69424 | 2.78E-72 |
| **FAM113B** | 0.728823 | 5.74E-83 | **SLA** | 0.633854 | 7.28E-57 |
| **FAM179A** | 0.72671 | 2.88E-82 | **SLA2** | 0.770834 | 2.07E-98 |
| **FAM26F** | 0.612769 | 2.95E-52 | **SLAMF1** | 0.707075 | 4.59E-76 |
| **FAM78A** | 0.671437 | 4.97E-66 | **SLAMF6** | 0.777798 | 2.72E-101 |
| **FASLG** | 0.631434 | 2.56E-56 | **SLAMF7** | 0.636567 | 1.75E-57 |
| **FCRL3** | 0.66051 | 3.14E-63 | **SLFN12L** | 0.693452 | 4.68E-72 |
| **FERMT3** | 0.604637 | 1.43E-50 | **SNX20** | 0.65403 | 1.27E-61 |
| **FGD2** | 0.686209 | 5.15E-70 | **SP140** | 0.749688 | 2.99E-90 |
| **FMNL1** | 0.73744 | 6.85E-86 | **SPN** | 0.613146 | 2.46E-52 |
| **FNBP1** | 0.646028 | 1.08E-59 | **ST8SIA1** | 0.641767 | 1.09E-58 |
| **FOXP3** | 0.628622 | 1.09E-55 | **STAT4** | 0.667687 | 4.69E-65 |
| **GAB3** | 0.669031 | 2.10E-65 | **TAP1** | 0.60382 | 2.10E-50 |
| **GBP2** | 0.613229 | 2.36E-52 | **TBC1D10C** | 0.771587 | 1.02E-98 |
| **GBP5** | 0.696232 | 7.43E-73 | **THEMIS** | 0.614692 | 1.16E-52 |
| **GFI1** | 0.838652 | 6.44E-132 | **TIGIT** | 0.777714 | 2.95E-101 |
| **GMIP** | 0.674666 | 7.00E-67 | **TMC8** | 0.839579 | 1.76E-132 |
| **GPR132** | 0.648213 | 3.26E-60 | **TMEM155** | 0.610363 | 9.43E-52 |
| **GPR171** | 0.75575 | 1.67E-92 | **TRAF1** | 0.745852 | 7.37E-89 |
| **GPR174** | 0.662759 | 8.52E-64 | **TRAF3IP3** | 0.814024 | 3.61E-118 |
| **GPR18** | 0.676983 | 1.69E-67 | **TRAF5** | 0.692118 | 1.13E-71 |
| **GPSM3** | 0.668497 | 2.89E-65 | **TRAT1** | 0.696858 | 4.89E-73 |
| **GRAP2** | 0.640385 | 2.29E-58 | **TSPAN32** | 0.640994 | 1.66E-58 |
| **GTSF1L** | 0.701173 | 2.67E-74 | **TTC16** | 0.619208 | 1.26E-53 |
| **GVIN1** | 0.622566 | 2.36E-54 | **TTC24** | 0.765514 | 2.82E-96 |
| **GZMA** | 0.656403 | 3.32E-62 | **TTN** | 0.637548 | 1.04E-57 |
| **GZMK** | 0.672221 | 3.09E-66 | **UBA7** | 0.60653 | 5.86E-51 |
| **HCLS1** | 0.602766 | 3.45E-50 | **UBASH3A** | 0.711738 | 1.72E-77 |
| **HLA_DOB** | 0.652057 | 3.85E-61 | **VAMP1** | 0.651508 | 5.23E-61 |
| **HMHA1** | 0.681777 | 8.55E-69 | **VAV1** | 0.632105 | 1.81E-56 |
| **HSF5** | 0.60361 | 2.32E-50 | **WAS** | 0.701069 | 2.87E-74 |
| **ICOS** | 0.762251 | 5.38E-95 | **WIPF1** | 0.649274 | 1.81E-60 |
| **IFNG** | 0.623233 | 1.69E-54 | **ZAP70** | 0.812019 | 3.85E-117 |
| **IKZF1** | 0.670134 | 1.09E-65 | **ZBED2** | 0.633847 | 7.30E-57 |
| **IL10RA** | 0.74718 | 2.45E-89 | **ZBTB32** | 0.657387 | 1.89E-62 |
| **IL12RB1** | 0.724675 | 1.34E-81 | **ZNF101** | 0.659533 | 5.52E-63 |
| **IL16** | 0.68096 | 1.43E-68 | **ZNF80** | 0.750273 | 1.82E-90 |
| **IL18BP** | 0.62099 | 5.19E-54 | **ZNF831** | 0.770738 | 2.27E-98 |
| **IL2RB** | 0.630511 | 4.13E-56 |  |  |  |

**Table S2 Comparison of CIBERSORT immune cell fractions**

| **Immune cell type** | **fraction in % of all infiltrating immune cells** | | ***p* value** |
| --- | --- | --- | --- |
|  | **CTLA4_high** | **CTLA4_low** |  |
| B cells naive | 2.785 | 5.222 | <0.001 |
| B cells memory | 0.087 | 0.090 | 0.038 |
| Plasma cells | 4.808 | 5.840 | 0.002 |
| T cells CD8+ | 19.207 | 8.275 | <0.001 |
| T cells CD4+ memory resting | 15.694 | 17.509 | 0.041 |
| T cells CD4+ memory activated | 0.248 | 0.013 | <0.001 |
| T cells follicular helper | 3.945 | 2.376 | <0.001 |
| T cells regulatory (Tregs) | 3.840 | 2.809 | <0.001 |
| T cells gamma delta | 1.001 | 0.098 | <0.001 |
| NK cells resting | 2.415 | 4.312 | <0.001 |
| NK cells activated | 1.327 | 1.935 | 0.002 |
| Monocytes | 4.239 | 5.504 | <0.001 |
| Macrophages M1 | 8.097 | 5.991 | <0.001 |
| Macrophages M2 | 24.223 | 28.286 | <0.001 |
| Dendritic cells resting | 0.126 | 0.216 | 0.001 |
| Dendritic cells activated | 0.185 | 0.479 | 0.005 |
| Mast cells resting | 3.214 | 6.071 | <0.001 |
